# Cross‐sectional investigation of the distribution characteristics and prognostic significance of lateral lymph nodes in patients with rectal cancer

**DOI:** 10.1002/cam4.70170

**Published:** 2024-09-23

**Authors:** Xuyang Yang, Yang Zhang, Zixuan Zhuang, Hanjiang Zeng, Tong Zhang, Xiangbing Deng, Wenjian Meng, Ziqiang Wang

**Affiliations:** ^1^ Colorectal Cancer Center, Department of General Surgery West China Hospital, Sichuan University Chengdu China; ^2^ Department of Radiology West China Hospital, Sichuan University Chengdu China

**Keywords:** computed tomography, distribution characteristics, lateral lymph nodes, prognostic significance, rectal cancer

## Abstract

**Background:**

Information about the distribution characteristics and prognostic significance of lateral lymph nodes (LLNs) on primary computed tomography (CT) scan in rectal cancer patients is lacking.

**Methods:**

Between January 2013 and December 2016, patients with pathologically proved rectal cancer and pretreatment abdominal enhanced CT in our department were screened. We firstly redivided LLNs into seven categories based on their locations. Then, the number and distribution of all measurable LLNs and the characteristics of the largest LLN in each lateral compartment were recorded. Furthermore, we investigated the long‐term outcomes in patients with different LLN characteristics and LLN risk scoring.

**Results:**

A total of 572 patients were enrolled in this study. About 80% of patients had measurable LLNs, and most patients developed measurable LLNs in the obturator cranial compartment. Lateral local recurrence (LLR) was observed in 20 patients, which accounted for 83.3% of the local recurrence (LR). Patients with the largest LLN short‐axis diameter >10 mm had a poor prognosis, which was similar to that in patients with simultaneous distant metastasis (SDM). Patients with LLN risk scoring ≥2 had a worse prognosis than those with LLN risk scoring <2, while better than those with SDM.

**Conclusion:**

This study suggests that LLR is the main locoregional recurrence pattern. Most rectal cancer patients have measurable LLNs on primary CT scan. However, patients with enlarged LLNs <10 mm or LLN risk scoring <2 still have a significantly better prognosis than patients with SDM, which indicated the potential value of locoregional treatment for these LLNs.

## Graphical abstract

Lateral local recurrence (LLR) is the main locoregional recurrence pattern, and most rectal cancer patients have measurable lateral lymph nodes (LLNs) on primary CT scan. Patients with the largest LLN short‐axis diameter >10 mm had a poor prognosis. Patients with LLN risk scoring ≥2 had a worse prognosis.

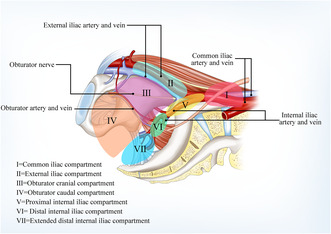

## 1 INTRODUCTION

Along with standard total mesorectal excision (TME) and neoadjuvant chemoradiotherapy (nCRT) wide applications, the rate of local recurrence (LR) in rectal cancer, especially central recurrence due to circumferential margin involvement, is decreasing to 5%–10%.^1^, ^2^, ^3^, ^4^, ^5^ Meanwhile, local lateral recurrence (LLR) due to lateral lymph node metastasis (LLNM) becomes the main locoregional recurrence pattern,^6^, ^7^ and obtains more attention for the severe complication, low salvage likelihood, and eventual death. However, the treatment of LLNM remains controversial.

In recent years, there has been a tendency toward that the treatment of LLNM depending on the lateral lymph nodes (LLNs) risk stratification based on the characteristics of primary tumor and LLNs.^8^ In detail, lateral lymph node disease was classified into three categories including low‐risk group (cT1/T2/early T3 without LLNM), moderate‐risk group (cT3/T4 without LLNM), and high‐risk group (clinically suspected LLNM). The treatment strategies were decided by risk classification. TME surgery only is enough for patients with low risk. nCRT or lateral lymph node dissection (LLND) could control LLR in patients with moderate risk, while, for patients with high risk, nCRT combined with LLND should be used to control LLR. One international study (LaNoReC trial) is performed to investigate the optimal treatment strategy, which is based on the LLNs short‐axis.^9^ In patients with LLNs ≥7 mm on primary MRI and still >4 mm on restaging MRI, the 5‐year LLR rate was more than 50%, which indicated the necessity of LLND, while, for other patients with LLNs ≤4 mm on restaging MRI, LLND could not be recommended. This risk‐stratified treatment strategy requires a comprehensive investigation of LLN characteristics in the whole rectal cancer patient population. Some previous studies had investigated the relationship between the LLN characteristics on MRI scans and long‐term outcomes.^9^, ^10^ As we know, MRI was widely used to evaluate primary tumor stage including T stage and N stage. However, MRI scan still has some drawbacks. On one hand, in clinical practice, MRI scan takes a long time, and it takes more than 30 minutes if the MRI scan ranged from the common iliac to the anus. Thus, usually, the routine pelvic MRI scan range did not include the common iliac compartment, the external iliac compartment, and the proximal internal iliac compartment. Therefore, it is difficult for MRI‐based studies to adequately characterize the LLNs in the whole population. On the other hand, due to the presence of an interlayer gap in MRI scan, LLNs (especially <5 mm) are easily missed. Therefore, it is difficult for MRI‐based studies to adequately characterize the LLNs in the whole population. Additionally, in China, high‐resolution MRI was not widely used in every hospital, while MDCT was more commonly applied in primary hospitals in economically underdeveloped areas. Furthermore, some authors had proved that, for predicting LLNM, MDCT had a sensitivity of 0.97 and the areas under the curve (AUCs) were 0.88. These results demonstrated that the accuracy of MDCT was satisfactory for predicting LLNM.^11^

In this cross‐sectional study, we firstly investigate the distribution characteristics of LLNs on multidetector‐row spiral computer tomography (MDCT) scan in the whole rectal cancer patient population. Furthermore, we also investigate the prognostic significance of patients with different LLN stratification.

## 2 MATERIALS AND METHODS

### 2.1 Study participants and patient selection

Between January 2013 and December 2016, patients who were admitted to our department were selected from the prospective database of colorectal cancer in our hospital. This retrospective study was approved by Ethics Committee on Biomedical Research, West China Hospital of Sichuan University, and this study has been retrospectively registered with the Clinical Trials (PID 209559).

On the whole, the eligibility criteria were as follows: patients with pathologically proved rectal cancer; the distance of the tumor from the anal margin ≤12 cm; and patients with pretreatment abdominal enhanced MDCT. Patients with recurrent rectal cancer, multiple primary cancer, or incomplete medical records were excluded. When investigating the LLN characteristics in the whole population, all patients with pathologically proved rectal cancer were included, although some patients did not accept surgery or curative surgery due to various reasons. When investigating the prognostic significance of LLN characteristics, only consecutive patients with curative resection were included, while patients with SDM were excluded.

### 2.2 Computed tomography protocol

In our study, CT scan protocol was standardized, and abdomen phantom was performed on SOMATOM Definition Flash (Siemens Healthcare, Erlangen, Germany). The CT scan parameters were identical to the manufacturer's standard: slice thickness 2 mm, pitch 0.992:1; tube current 200–450 mA, tube voltage, 100/120 kVp; rotation speed: 0.5 s/rot; and ASIR‐V: 20%. In this study, all patients received MDCT of the abdomen with contrast media containing a high concentration of iodine (iopromide 370 mg/mL, Bayer AG, Germany; volume, 1.5–2.0 mL/kg of body weight) at a rate of 2–3 mL/s. CT scan was performed before and after intravenous administration of contrast material (30–35 s arterial phase and 60–70s portal venous phase).

### 2.3 Reassessment of MDCT

**FIGURE 1:**
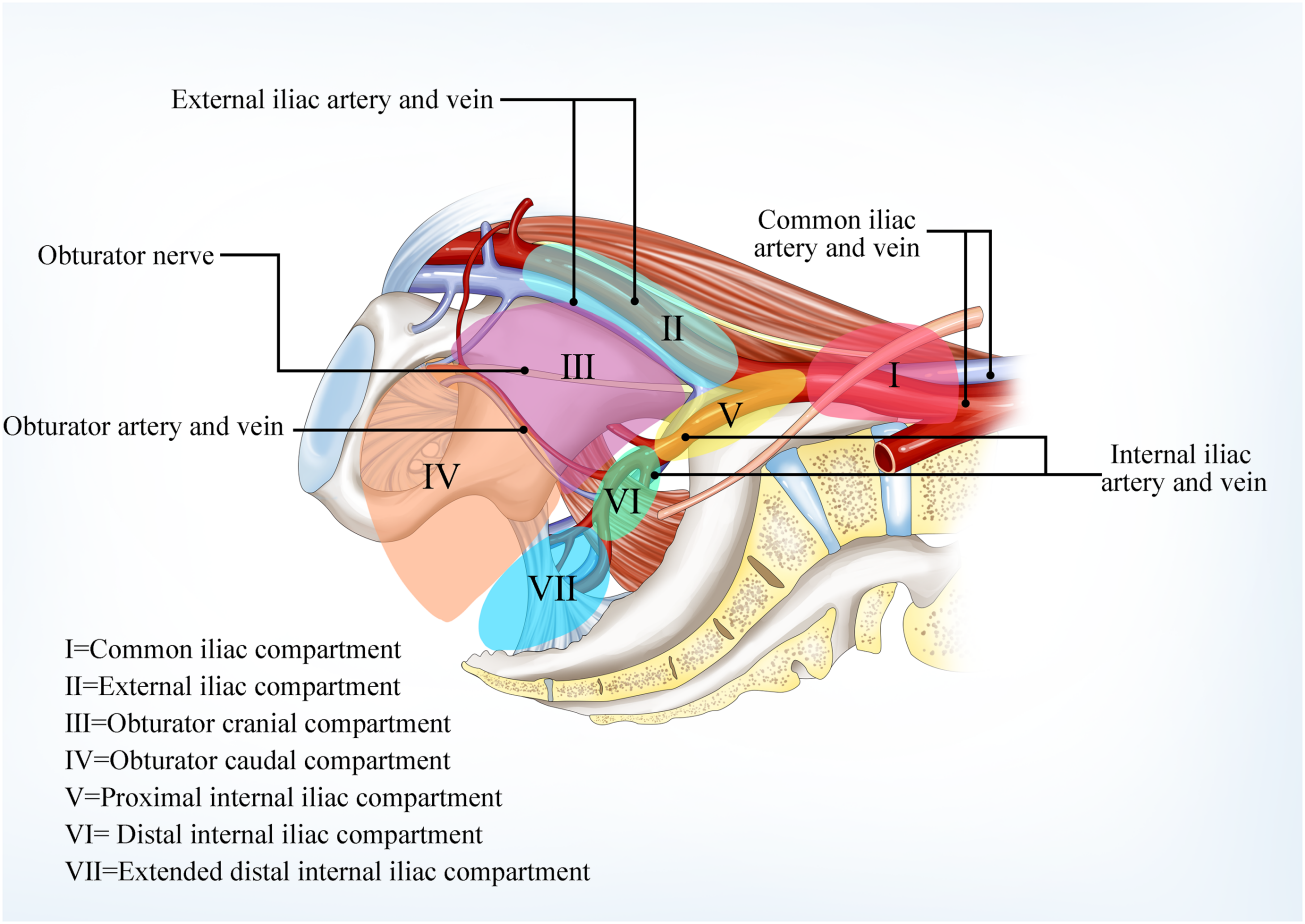
The schematic diagram showed the seven lateral compartments. (I) (light red), common iliac compartment; (II) (sky blue), external iliac compartment; (III) (purple), obturator cranial compartment; (IV) (light orange), obturator caudal compartment; (V) (yellow): Proximal internal iliac compartment; (VI) (light green): Distal internal iliac compartment; (VII) (blue): Extended distal internal iliac compartment.

**FIGURE 2:**
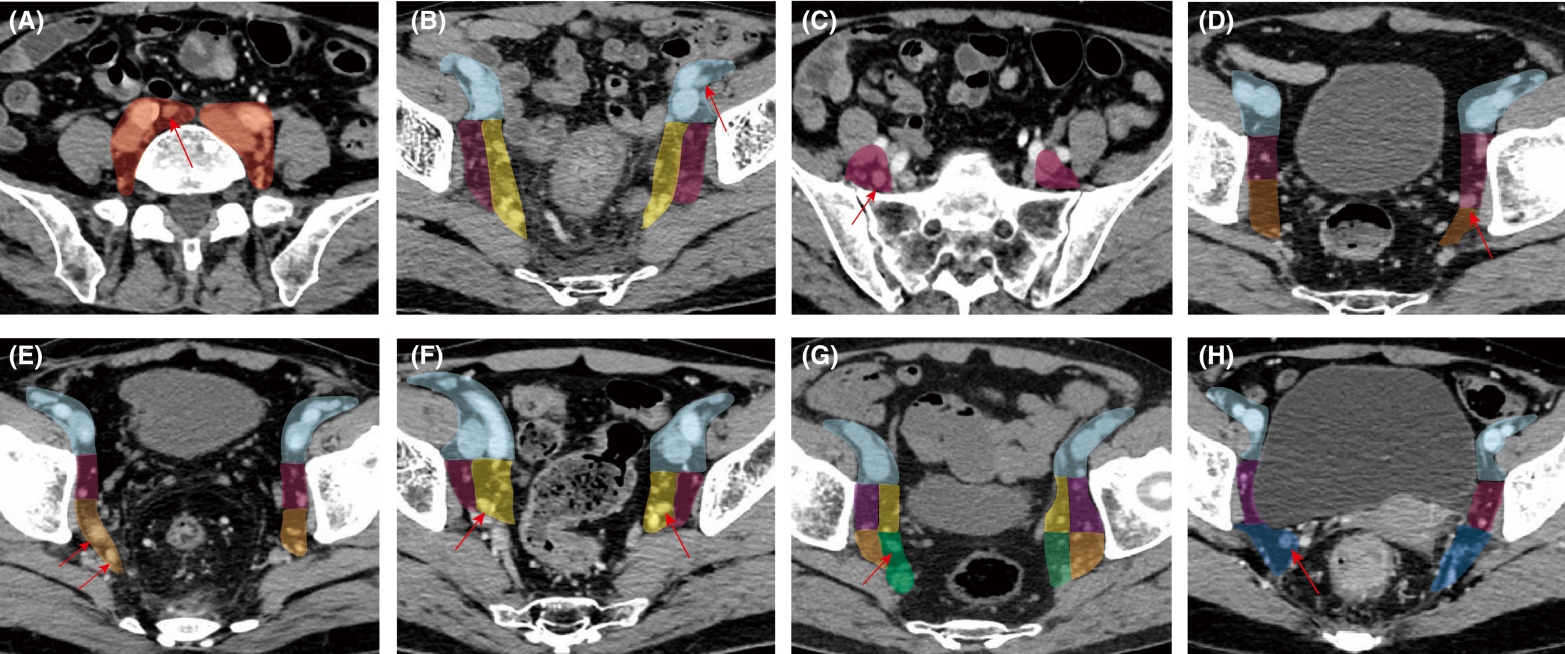
CT scan showed LLNs located the seven lateral compartments. (A) (light red), common iliac compartment; (B) (sky blue), external iliac compartment; (C, D) (purple), obturator cranial compartment; (E) (light orange), obturator caudal compartment; (F) (yellow): Proximal internal iliac compartment; (G) (light green): Distal internal iliac compartment; (H) (blue): Extended distal internal iliac compartment. Red arrow indicated LLNs.

The pretreatment MDCT scan was re‐evaluated by two experienced radiologists (Zeng HJ and Zhang T) who were blind to clinicopathological information. Differences in the radiologic conclusions were resolved by discussion. As shown in Figures 1 and 2, to more comprehensively investigate the distribution characteristics of LLNs in detail, we redivided LLNs into seven categories according to their locations and the Japanese Society for Cancer of the Colon and Rectum (JSCCR) guideline.^12^ The common iliac lymph nodes (LNs) and external iliac LNs were still divided according to the JSCCR guideline (Figure 1I,II; Figure 2A,B). Based on the relationship between LNs and the posterior margin of the obturator artery, the obturator LNs were classified into obturator cranial LNs (LNs located on the cranial side of the obturator artery) and obturator caudal LNs (LNs located on the caudal side of the obturator artery) (Figure 1III,IV; Figure 2C–E). The obturator compartment and the internal iliac compartment were divided by the imaginary curve that connected the lateral borders of the visceral branches of the internal iliac vessels. The plane of this imaginary curve is equivalent to the plane in which the bladder hypogastric fascia is located. The medial region is the internal iliac region, and the lateral region is the obturator region. The internal iliac LNs were further classified into three groups: the proximal internal iliac LNs, the distal internal iliac LNs, and the extended distal internal iliac LNs. Given that the superior gluteal artery is easily observed on CT scan, we use this artery to identify the proximal internal iliac LNs and the distal internal iliac LNs (Figure 1V,VI; Figure 2*F*,G). The extended distal internal iliac compartment refers to the internal iliac compartment below the inferior border of the piriformis muscle, which contains the pelvic plexus and neurovascular bundles associated with erection. This area is not covered by traditional LLND (Figure 1VII; Figure 2H).

The number and distribution of all measurable LLNs were carefully detected, and the characteristics of the largest LLN in each lateral compartment were recorded. As described by previous studies,^13^ the characteristics of the largest LLN were recorded as followed: (1) the short‐axis and long‐axis diameter; (2) LLN shape including regular morphology (round) and irregular morphology (ovoid and elongated); (3) the border features including smooth, lobulated, spiculated, and indistinct; (4) texture (homogeneous or heterogeneous appearance); and (5) CT signal intensity. The LLN malignant features included the short‐axis diameter >5 mm, irregular morphology, spiculated border, and heterogeneous appearance. In this study, we try to establish a comprehensive risk scoring system using the largest LLN characteristics on CT scan to stratify the risk of LLNM, and then, we further identify the prognostic significance of this risk scoring system. We define if the largest LLN with a short‐axis diameter <3 mm would receive 0 points, 3–5 mm 1 point, 5–7 mm 2 points, 7–10 mm 3 points; and >10 mm 4 points; one point was added for irregular morphology, spiculated border, or heterogeneous appearance.

### 2.4 Clinical staging and treatment strategy

In this study, mid/low rectal cancer was defined as the distance between the lower edge of the tumor and the anal edge ≤12 cm. Digital rectal examination, rigid sigmoidoscopy, and pelvic magnetic resonance imaging were combined to evaluate tumor height. TNM staging is based on the UICC's TNM staging system.^14^ High‐resolution pelvic MRI and transrectal ultrasound were routinely combined to assess the T staging. In this study, the largest LLN was defined as LLN with the largest short‐axis diameter. Between 2013 and 2016, in our clinical practice work, the diagnostic criterion of clinically suspected LLNM was the short‐axis diameter of the largest LLN ≥5 mm before any treatment. Locally advanced rectal cancer was defined as rectal cancer with positive lymph nodes or cT3/4 stage. In general, the treatment strategy was decided by the multidisciplinary team meeting (MDT) as descripted in our previous studies.^15^ The preoperative regimens included short‐term radiotherapy (25Gy) and long‐term chemoradiotherapy based on 5‐FU (45–50.4 Gy), and the radiotherapy area conventionally covered the lateral region. Surgery was usually performed 1 week after short‐term radiotherapy, and 6–8 weeks after long‐term chemoradiotherapy. For patients with clinically suspected LLNM before treatment, standard TME plus LLND was performed no matter whether these patients accepted nCRT or not. Only when both sides had clinically suspected LLNM, bilateral LLND was considered. Postoperative adjuvant chemotherapy was considered when patients developed stage III or stage II with high‐risk factors according to the pathological results.

### 2.5 Follow‐up

The follow‐up principle was consistent with our previous study.^16^ Briefly, physical examination including digital rectal examination and serum tumor markers were reviewed every 3 months for the first 2 years, every 6 months for the next 3 years, and annually beyond 5 years. Chest CT and enhanced abdominal CT scans were recommended every 6 months for the first 2 years and annually since then.

Local recurrence (LR) included tumor recurrence in any part of the anastomotic site, pelvic cavity, and perineum. Lateral local recurrences (LLR) refer to tumor regrowth in one of the LLN basins mentioned above. The overall survival (OS) was defined as the time between surgery and death, or the final follow‐up time. The local recurrence‐free survival (LRFS) was defined as the time from the end of surgery to local recurrence, or to the final follow‐up time. The lateral local recurrence‐free survival (LLRFS) was defined as the time between surgery and lateral local recurrence, or the final follow‐up time. The distant metastasis‐free survival (DMFS) was defined as the time from the end of surgery to distant metastasis‐free survival, or the final follow‐up time.

### 2.6 Statistical analyses

R software (version 4.0.5) and SPSS software (version 22.0) were used for the statistical analyses. Continuous variables were shown as mean with standard deviation (SD), and differences between the two groups were tested using Student's *t‐*test. Categorical variables were expressed by absolute number with percentage, and differences between two groups were tested by Pearson's chi‐squared test or the Fisher's exact test. The 5‐year OS and DFS were evaluated using the Kaplan–Meier method with the log‐rank test. Kaplan–Meier survival curves were constructed using R (R function: survfit, ggsurvplot; package: survival, survminer). Differences were considered statistically significant if *p*‐values were <0.05.

## 3 RESULTS

Initially, a total of 572 patients with pathology‐proved mid/low rectal cancer were collected, including 35 (6.1%) patients without surgery or curative surgery and 50 (8.7%) patients with simultaneous distant metastasis (SDM) and accepting curative primary tumor resection. The clinicopathological characteristics of patients included were showed in Table 1. The majority were male patients (62.1%), and the mean age was 60.3 years. Most patients (74.3%) had BMI <24 kg/m^2^. The mean tumor distance from anal verge was 6.3 cm. Most patients had a clinical T3 (60.5%) or clinical N0 tumor (55.9%). About 79% of patients didn't receive any nCRT and underwent primary surgery. There were 51 (9.5%) patients who accepted short‐term radiotherapy, and those patients were derived from our RCT study.^17^ Additionally, there were 46 (8.6%) patients accepted long‐course nCRT, and 18 (3.4%) patients accepted neoadjuvant chemotherapy. A total of 74.3% of patients underwent low anterior resection (LAR), and more than 52% of patients received postoperative adjuvant therapy. In this period, due to the lack of attention and technically challenging, there were only 18 (3.4%) patients underwent LLND. Only two patients (0.4%) had pathological LLNM. Similar to the clinical stage, most patients had a pathological T3 (51.4%) or pathological N0 tumor (64.2%).

**TABLE 1:** The clinicopathological characteristics of patients with rectal cancer.

Characteristics	Number (*n* = 572)	Score <2 (*n* = 228)	Score ≥2 (*n* = 259)	SDM (*n* = 50)	*p*‐value
Sex, *n* (%)					
Male	355 (62.1)	146 (64.0)	150 (57.9)	36 (72.0)	0.119
Female	217 (37.9)	82 (26.0)	109 (42.1)	14 (28.0)	
Age, mean ± SD, year	60.3 ± 12.9	61.4 ± 12.0	59.6 ± 13.2	56.7 ± 13.0	0.039
BMI, mean ± SD, kg/m^2^					
<25	425 (74.3)	176 (77.2)	182 (70.3)	40 (80.0)	0.138
≥25	147 (25.7)	52 (22.8)	77 (29.7)	10 (20.0)	
Tumor distance from AV, mean + SD, cm	6.3 ± 2.9	6.4 ± 3.0	6.0 ± 2.9	7.2 ± 2.9	0.022
cT, *n* (%)					
T1	27 (4.7)	14 (6.1)	12 (4.6)	1 (2.0)	0.003
T2	118 (20.6)	59 (25.9)	50 (19.3)	2 (4.0)	
T3	346 (60.5)	138 (60.5)	160 (61.8)	37 (74.0)	
T4	81 (14.2)	17 (7.5)	37 (14.3)	10 (20.0)	
cN, *n* (%)					
N0	320 (55.9)	150 (65.8)	147 (56.8)	17 (34.0)	
N1	188 (32.9)	62 (27.2)	85 (32.8)	26 (52.0)	
N2	64 (11.2)	16 (7.0)	27 (10.4)	7 (14.0)	
nCRT, *n* (%)					
Short‐course RT	51 (9.5)	21 (9.2)	28 (10.8)	28 (56.0)	0.000
Long‐course CRT	46 (8.6)	13 (5.7)	27 (10.4)	2 (4.0)	
Chemotherapy	18 (3.4)	1 (0.4)	3 (16.7)	6 (12.0)	
Primary surgery	422 (78.6)	193 (84.6)	201 (77.6)	14 (28.0)	
Adjuvant therapy, *n* (%)	279 (52.0)	104 (45.6)	138 (53.3)	37 (74.0)	0.001
Type of procedure, *n* (%)					
LAR	399 (74.3)	176 (77.2)	187 (72.2)	36 (72.0)	0.589
ELAPE or Miles	85 (15.8)	28 (12.3)	48 (18.5)	9 (18.0)	
ISR	31 (5.8)	15 (6.6)	14 (5.4)	2 (4.0)	
Hartmann	22 (4.1)	9 (3.9)	10 (3.9)	3 (6.0)	
LLND procedure	18 (3.4)	1 (0.4)	15 (5.8)	2 (4.0)	0.005
pT, *n* (%)					
T0	14 (2.6)	7 (3.1)	7 (2.7)	0 (0.0)	0.000
T1	40 (7.4)	21 (9.2)	18 (6.9)	1 (2.0)	
T2	153 (28.5)	74 (32.5)	76 (29.3)	3 (6.0)	
T3	276 (51.4)	110 (48.2)	136 (52.5)	30 (60.0)	
T4	54 (10.1)	16 (7.0)	22 (8.5)	16 (32.0)	
pN, *n* (%)					
N0	345 (64.2)	160 (70.2)	168 (64.9)	17 (34.0)	0.000
N1	148 (27.6)	49 (21.5)	72 (27.8)	27 (54.0)	
N2	44 (8.2)	19 (8.3)	19 (7.3)	6 (12.0)	
Patients with mesorectal lymph nodes metastasis, *n* (%)	169 (31.5)	60 (26.3)	78 (30.1)	31 (62.0)	0.000
Patients with lateral lymph nodes metastases, *n* (%)	2 (0.4)	0 (0.0)	0 (0.0)	2 (4.0)	0.000
Number of mesorectal lymph nodes harvested, mean ± SD	12.1 ± 7.2	11.9 ± 6.8	12.2 ± 7.6	12.3 ± 7.1	0.901
Number of metastatic lymph nodes in mesorectum, mean ± SD	0.9 ± 2.0	0.7 ± 1.8	0.9 ± 2.3	1.5 ± 1.9	0.077
Number of lateral lymph nodes harvested, mean ± SD	14.8 ± 7.2	12.0	15.3 ± 7.7	11.0 ± 2.8	0.677
Number of metastatic lateral lymph nodes, mean ± SD	0.2 ± 0.5	0.0	0.0	1.5 ± 0.7	0.000

Abbreviations: AV, anal verge; BMI, body mass index; CRT, chemoradiotherapy; ELAPE, extralevator abdominoperineal excision; ISR, internal sphincter resection; LAR, low anterior resection; nCRT, neoadjuvant chemoradiotherapy; RT, radiotherapy; SD, standard deviation.

**FIGURE 3:**
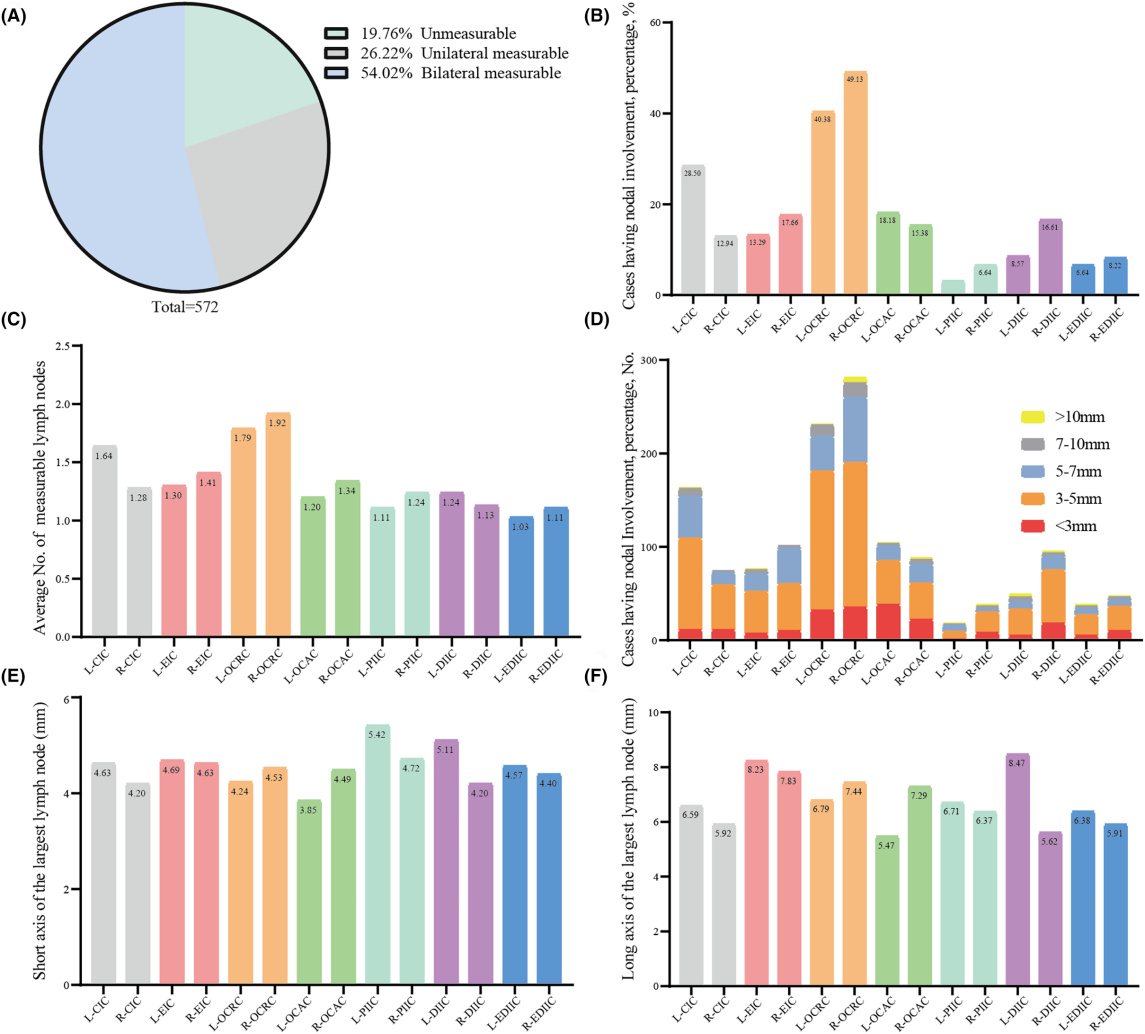
The distribution compartments of all measurable lateral lymph nodes. (A) The proportion of patients with unmeasurable, unilateral measurable, and bilateral measurable lateral lymph nodes. (B) The proportion of patients with different compartment lateral lymph nodes. (C) The average lateral lymph nodes observed in each compartment. (D) The proportion of patients with different compartment lateral lymph nodes divided by short axis. (E) The short axis of the largest lymph nodes in short axis in different compartments. (F) The long axis of the largest lymph nodes in short axis in different compartments.

The number of all measurable LLNs in the lateral compartments was recorded, and the distribution characteristics of these LLNs were revealed in Table S1 and Figure 3. Interestingly, as shown in Figure 3A, we found that on primary CT scan, the majority (80%) had measurable LLNs (26.22% unilateral and 54.02% bilateral), while the minority (19.76%) had unmeasurable LLNs. As expected, most measurable LLNs were located in the obturator cranial compartment. A total of 231 (40.38%) patients and 281 (49.13%) patients had left and right obturator cranial LNs, respectively. Notably, LLNs located in the distal internal iliac compartment and extended distal internal iliac compartment usually were not easily to be completely resected. The proportion of patients with left and right distal internal iliac LNs was 8.57% and 16.61%, respectively, and patients with left and right extended distal internal iliac LNs was 6.64% and 8.22%, respectively (Figure 3B). The average number of LLNs in the obturator cranial compartment was more than that in other compartments (1.79 on the left side, and 1.92 on the right side, respectively). The number of LLNs in the left and right distal internal iliac compartment was 1.24 and 1.13, respectively, and in the left and right extended distal internal iliac compartment was 1.03 and 1.11, respectively (Figure 3C).

We further investigated the largest LLN short‐axis diameter distribution in the lateral compartments. With regard to lateral compartment distribution, we found the largest LLN short‐axis diameter with 3–5 mm and 5–7 mm accounted for the most in every single compartment (Table S2 and Figure 3D). The proportion of patients with the largest LLN short‐axis diameter with 3–5 mm and 5–7 mm in the left obturator cranial compartment was 64.5% and 16%, respectively, and in the right obturator cranial compartment was 55.2% and 24.9%, respectively. In the whole cohort, there were 20 (4.4%) patients with >10 mm LLN, 52 (11.3%) patients with 7–10 mm LLN, 158 (34.4%) patients with 5–7 mm LLN, 206 (44.9%) patients with 3–5 mm LLN, and 23 (5.0%) patients with 0–3 mm LLN (Table S3). Meanwhile, in the 50 patients with SDM, three (6%) patients had the largest LLN short‐axis diameter >10 mm. Then, the short‐axis and long‐axis diameter of the largest LLN were also recorded. LLNs in the left proximal internal iliac compartment and the left distal internal iliac compartment had the largest short‐axis diameter (5.42 ± 2.94) and long‐axis diameter (8.47 ± 11.34), respectively (Table S1 and Figure 3E,F), while LLNs in the left obturator caudal compartment had both the shortest short‐axis (3.85 ± 2.18) and long‐axis diameter (5.47 ± 2.99), respectively (Table S1 and Figure 3E,F).

**FIGURE 4:**
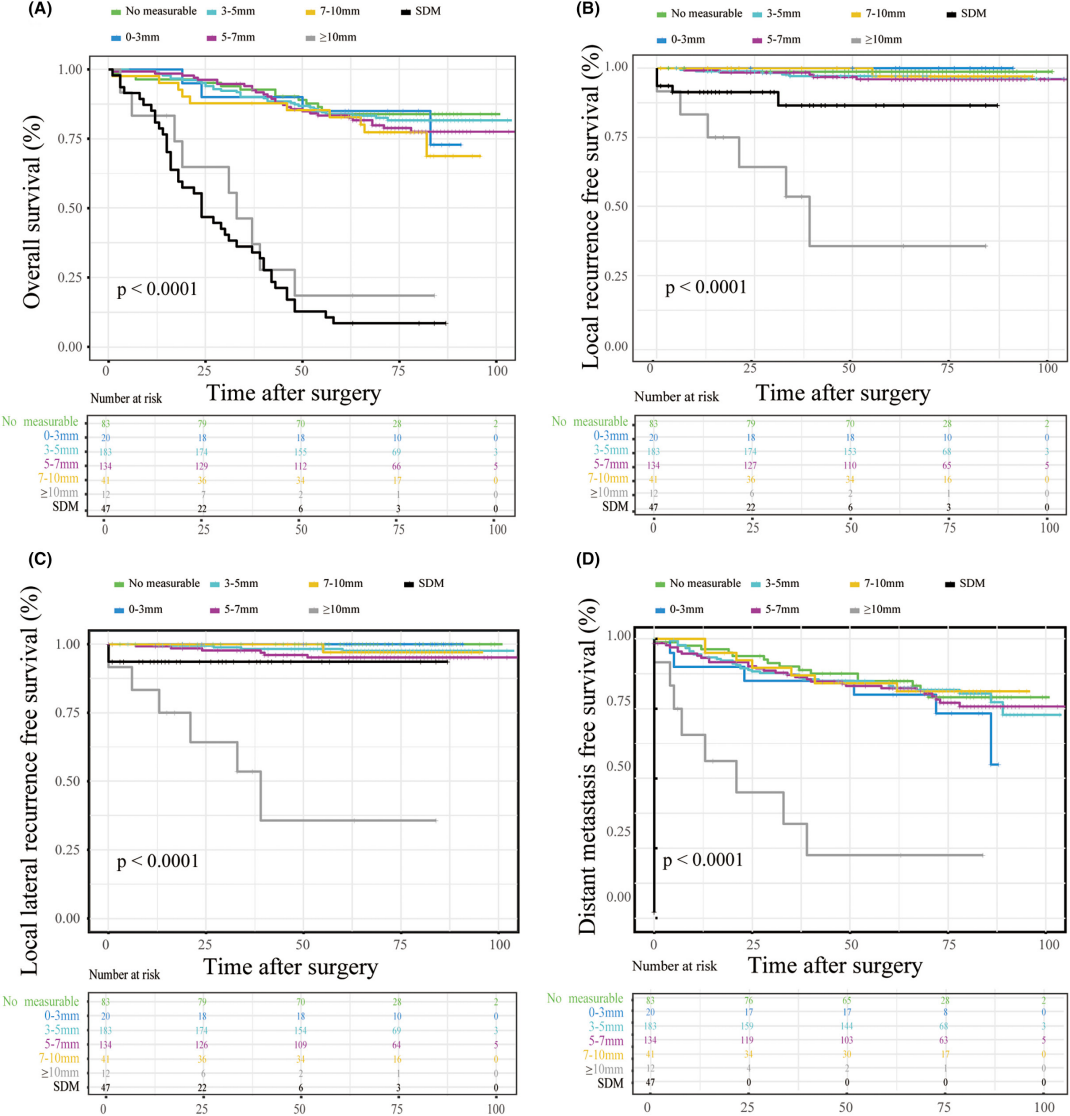
Survival outcomes of patients with different lateral lymph nodes short‐axis diameter (A) Overall survival; (B) Local recurrence‐free survival; (C) Lateral local recurrence‐free survival; (D) Distant metastasis‐free survival.

**FIGURE 5:**
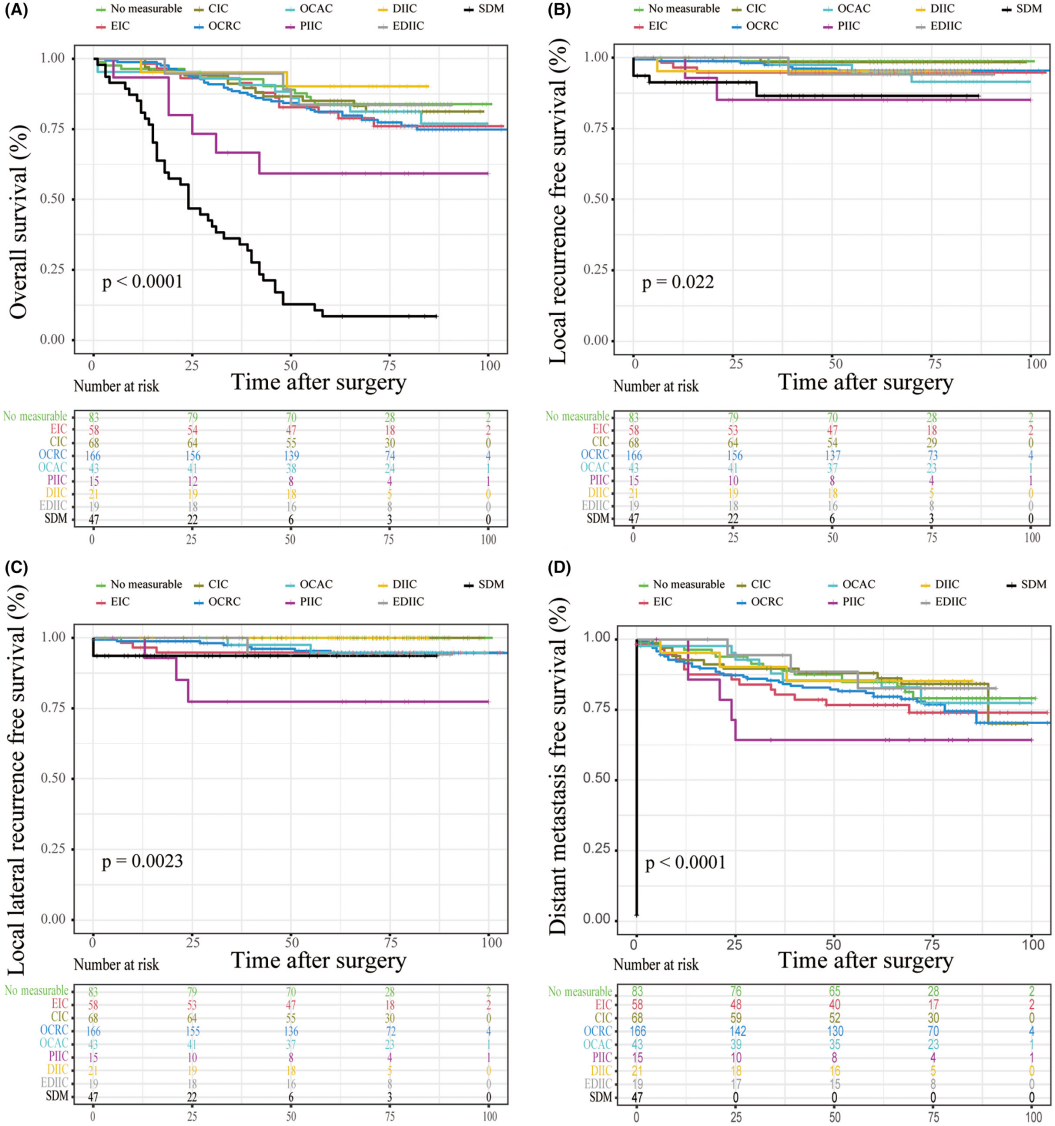
Survival outcomes of lymph nodes located in different lateral compartments. (A) Overall survival; (B) Local recurrence‐free survival; (C) Lateral local recurrence‐free survival; (D) Distant metastasis‐free survival.

To further investigate the prognostic significance of LLN characteristics, 52 (9.1%) patients were excluded for no operation or palliative surgery or being lost to follow‐up, and 520 (90.1%) patients including 47 (8.2%) patients with SDM were included to compare the long‐term outcomes with respect to different LLN characteristics (Figure 4). The median follow‐up duration was 75 months. LR was identified in 24 (4.6%) patients. Among them, LLR was observed in 20 patients, which accounted for 80% of the LR. Notably, in 20 patients with LLR, 5 (25%) patients with the largest LLN short‐axis diameter <5 mm on the primary CT scan. Additionally, a total of 99 (19%) patients who developed distant metastasis during follow‐up were also identified. With respect to OS, patients with different LLN sizes had different OS, and there was a tendency toward patients with larger LLN had worse OS (Figure 4A–D, Table S5). Patients with LLN short‐axis diameter >10 mm had the worst 5‐year OS (18.5%), which was comparable with that in patients with SDM (8.5%). However, no significant differences were observed between the other groups, and patients with LLN short‐axis diameter <10 mm had better 5‐year OS than patients with LLN >10 mm or SDM. With regard to LRFS and LLRFS, patients with LLN short‐axis diameter >10 mm had the worst 5‐year LRFS (35.7%) and LLRFS (35.7%). Of 50 patients with SMD, three patients developed LR, and one patient developed LLR. Additionally, there were no significant differences in 5‐year OS, LRFS, LLRFS, and DMFS among patients with 0–10 mm LLN. With respect to DMFS, patients with LLN short‐axis diameter >10 mm had the highest risk of distant metastasis, and the 5‐year DMFS was 22.5%. Additionally, we also investigate the relationship between different LLN locations and oncological outcomes. We found that lymph nodes located in the proximal internal iliac compartment were related to poor outcomes, including OS, LRFS, LLRFS, and DMFS (Figure 5A–D).

**FIGURE 6:**
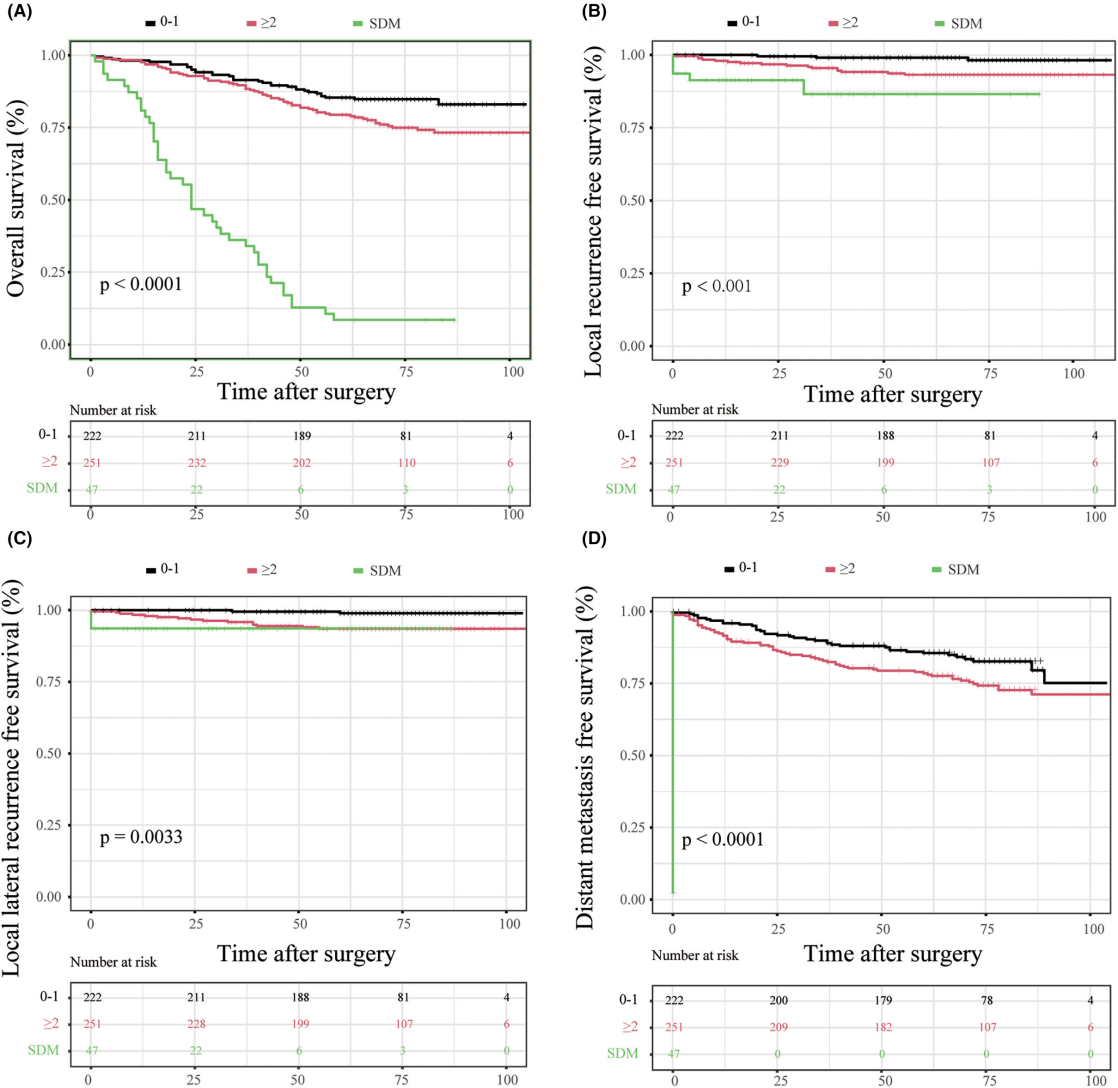
Survival outcomes of patients with different lateral lymph nodes risk scoring (A) Overall survival; (B) Local recurrence‐free survival; (C) Lateral local recurrence‐free survival; (D) Distant metastasis‐free survival. SDM, simultaneous distant metastasis.

The other imaging characteristics of the largest LLN were shown in Table S4. The proportion of unilateral and bilateral distribution of the largest LLN was 32.7% and 67.3%, respectively. The frequencies of the LLN with irregular morphology, heterogeneous texture, and spiculated margin were 35.5%, 24.4%, and 13.5%, respectively. As described in the methods section, we established a 7‐point risk scoring system to stratify the risk of LLNM, and patients were divided into seven groups by their risk score. According to univariable and multivariable analysis, we identified LLN clinical risk scoring ≥2 as the optimal cutoff value for predicting long‐term outcomes (Figure 6; Table 2; Table S6). This means LLN with risk scoring ≥2 could be considered malignant LLN, which means LLN with short‐axis diameter >5 mm or with two malignant features or with short‐axis diameter 3‐5 mm and one malignant feature could be considered as clinically suspected LLNM. Overall, compared with patients with the LLN scoring <2, patients with the LLN scoring ≥2 had worse 5‐year OS, LRFS, LLRFS, and DMFS, while better than those patients with SDM (Figure 6A–D). The 5‐year OS in patients with the LLN scoring ≥2 and <2 was 79.4% and 85.4%, respectively. The 5‐year DFS in patients with the LLN scoring ≥2 and <2 was 76.1% and 85.1%, respectively. The 5‐year LR rate, LLR rate, and DMR rate in patients with the LLN scoring ≥2 were 6.8%, 6.5%, and 28.8%, while in patients with the LLN scoring <2 were 1.8%, 1.0%, and 24.9%. Patients with SDM had the worst 5‐year OS (8.5%), LRFS (86.5%), LLRFS (93.6%), and DMFS (0%). The clinicopathological characteristics of patients with the LLN scoring <2, ≥2, and SDM were shown in Table 1.

**TABLE 2:** Univariate and multivariate analyses of factors associated with the overall survival, local recurrence‐free survival, local lateral recurrence‐free survival, and distant metastasis‐free survival.

Variables	Overall survival	*p*‐value	Multivariate analysis	*p*‐value	Local recurrence‐free survival	*p*‐value	Multivariate analysis	*p*‐value	Local lateral recurrence‐free survival	*p*‐value	Multivariate analysis	*p*‐value	Distant metastasis‐free survival	*p*‐value	Multivariate analysis	*p*‐value
	Univariate analysis				Univariate analysis				Univariate analysis				Univariate analysis			
	HR (95% CI)		HR (95% CI)		HR (95% CI)		HR (95% CI)		HR (95% CI)		HR (95% CI)		HR (95% CI)		HR (95% CI)	
Sex: female/male	1.246 (0.818, 1.899)	0.306			1.113 (0.438, 2.828)	0.821			1.194 (0.442, 3.229)	0.727			1.012 (0.677, 1.513)	0.955		
Age ≥65 years	1.657 (1.106, 2.483)	0.014	1.913 (1.270, 2.886)	**0.002**	0.779 (0.296, 2.050)	0.613			0.703 (0.248, 1.995)	0.508			1.040 (0.695, 1.558)	0.848		
BMI ≥ 25 kg/m^2^	0.540 (0.320, 0.912)	0.021	0.508 (0.300, 0.861)	**0.012**	0.680 (0.226, 2.050)	0.493			0.790 (0.257, 2.422)	0.680			0.745 (0.467, 1.187)	0.215		
Tumor distance from AV ≤5 cm	1.369 (0.916, 2.046)	0.125			4.312 (1.431, 12.993)	0.009	4.850 (1.583, 14.861)	**0.006**	3.735 (1.218, 11.456)	0.021	4.128 (1.317, 12.941)	**0.015**	1.113 (0.752, 1.647)	0.594		
cT: 1,2/3,4	2.807 (1.533, 5.140)	0.001			2.125 (0.619, 7.293)	0.231			1.883 (0.541, 6.552)	0.320			4.192 (2.113, 8.318)	0.000		
cN: 0/1,2	1.618 (1.085, 2.414)	0.018			1.450 (0.589, 3.570)	0.418			1.4411 (0.556, 3.734)	0.453			1.893 (1.278, 2.803)	0.001		
Preoperative short‐course RT	1.184 (0.629, 2.230)	0.600			4.089 (1.534, 10.896)	0.005	2.876 (1.080, 7.658)	**0.035**	3.688 (1.281, 10.616)	0.016	2.597 (0.901, 7.485)	0.077	1.746 (1.018, 2.997)	0.043	1.520 (0.890, 2.598)	0.126
Preoperative long‐course CRT	1.482 (0.766, 2.868)	0.243			0.884 (0.115, 6.804)	0.906			0.9991 (0.128, 7.681)	0.993			1.339 (0.670, 2.675)	0.408		
Preoperative chemotherapy	0.000 (0.000, 1.346 × 10^220^)	0.967			0.045 (0.000, 180.476)	0.464			0.045 (0.000, 339.437)	0.497			1.017 (0.445, 2.321)	0.969		
Adjuvant therapy	0.891 (0.597, 1.330)	0.572			1.313 (0.528, 3.266)	0.557			1.374 (0.523, 3.611)	0.519			1.410 (0.944, 2.107)	0.093		
Sphincter‐preserving/Non‐sphincter‐preserving	1.894 (1.185, 3.027)	0.008	2.245 (1.392, 3.620)	**0.001**	1.561 (0.518, 4.704)	0.429			1.811 (0.590, 5.555)	0.299			1.350 (0.819, 2.225)	0.239		
pT: 0,1,2/3,4	1.928 (1.235, 3.008)	0.004	1.398 (0.871, 2.244)	0.165	6.448 (1.490, 27.912)	0.013	6.508 (1.430, 29.608)	**0.015**	5.754 (1.316, 25.163)	0.020	6.898 (1.559, 30.514)	**0.011**	2.589 (1.624, 4.126)	0.000	1.761 (1.076, 2.883)	**0.024**
pN: 0/1,2	2.447 (1.640, 3.652)	0.000	2.469 (1.599, 3.811)	**0.000**	2.553 (1.037, 6.287)	0.042	1.709 (0.674, 4.331)	0.259	2.055 (0.792, 5.330)	0.139			3.437 (2.309, 5.117)	0.000	2.797 (1.838, 4.258)	**0.000**
Number of mesorectal lymph nodes harvested ≥12	0.767 (0.511, 1.153)	0.203			0.655 (0.258, 1.664)	0.374			0.617 (0.228, 1.668)	0.341			0.986 (0.665, 1.462)	0.945		
Metastatic lymph nodes in mesorectum	2.208 (1.475, 3.305)	0.000			2.505 (1.107, 6.169)	0.046			1.961 (0.746, 5.156)	0.172			3.083 (2.082, 4.566)	0.000		
Malignant score ≥2	1.643 (1.081, 2.497)	0.020	1.659 (1.075, 2.559)	**0.022**	4.855 (1.415, 16.664)	0.012	4.055 (1.177, 13.974)	**0.027**	6.900 (1.578, 30.175)	0.010	6.012 (1.372, 26.349)	**0.017**	1.548 (1.031, 2.325)	0.035	1.436 (0.956, 2.157)	0.081

*Note*: Statistically significant *p*‐values in the multivariate analysis were shown in bold typeface.

Abbreviations: AV, anal verge; BMI, body mass index; CRT, chemoradiotherapy; ELAPE, extralevator abdominoperineal excision; HR, hazard ratio; ISR, internal sphincter resection; LAR, low anterior resection; nCRT, neoadjuvant chemoradiotherapy; RT, radiotherapy; SD, standard deviation.

## 4 DISCUSSION

The management of LLNM have always been controversial between the Eastern and Western countries. In recent decade years, with the wide application of nCRT and TME technique, pelvic central recurrence due to circumferential margin involvement or residual mesorectal tissue or lymph node involvement have been controlled well. Meanwhile, although the LLNM rate in rectal cancer is only 10%–25%, LLR caused by LLNM has become the main type of postoperative recurrence.^18^, ^19^, ^20^ In this study, we found that LLR accounted for 80% of LR, which was comparable with previous reports (54.6% to 82.7%).^6^, ^7^ Notably, 25% of patients with LLR had the largest LLN short‐axis diameter <5 mm, and those patients were easily missed diagnosis. Thus, our results further confirmed that LLR has become the major recurrence pattern in rectal cancer.^18^, ^19^, ^20^ Due to the low diagnostic accuracy of LLNM and the high rate of delayed diagnosis in LLR, patients with LLR had poor long‐term outcomes.^21^ Therefore, the management of LLNM is still challenging and should call more attention.

So far, there was still a lack of study on a comprehensive description of LLN characteristics in rectal cancer. Some previous studies had tried to assess the correlation between primary MRI detected LLN features and prognosis in rectal cancer patients. Given that small LLNs are likely to be missed on MRI scans, we performed the first study to investigate the LLN features on MDCT and their relationship with long‐term outcomes. One MRI‐based study demonstrated that even in pN0 patients, at least one visible LLN was detected in 25.1% of patients on primary MRI.^22^ The Lateral Node Study Consortium group had also reported that 58% of patients had visible LLNs on pretreatment MRI.^7^ Interestingly, the first main finding from this study was that approximately 80% of patients had measurable LLNs and more than half of patients had bilateral measurable LLNs on primary CT scan. These results demonstrated that CT scan can detect more LLNs, especially small LLNs and most patients had measurable LLNs before treatment. This finding strongly suggested that surgeons should pay more attention to LLNs to avoid missing diagnosis. Notably, 8% patients with distal internal iliac lymph node and 7% patients with extend distal internal iliac lymph node were identified, and more than 80% of those LLNs located in the two compartments were those with short‐axis diameter <5 mm. As we know, in routine clinical practice, LLNs located in the two compartments were easily ignored on imaging. On the contrary, it is technically challenging to completely dissect those LLNs in the two compartments. Our results revealed that lymph nodes located in the internal iliac compartment had a poor prognosis. However, one Japanese study reported that the 5‐year DFS tended to be poorer of the obturator LLN group than those of the internal iliac LLN group, but the difference was not significant (*p* = 0.075).^23^ In contrast, a multicenter study suggested that compared with other compartments, internal iliac lymph nodes were less likely to respond to neoadjuvant therapy and associated with an increased LLR rate.^24^ In a study conducted with a large sample size at the Japanese National Cancer Center Hospital, involving 469 rectal cancer patients who underwent lateral lymph node dissection, it was found that local recurrence occurred most frequently in the pelvic plexus and internal iliac area (6.6%), compared to the markedly lower rate in the obturator area (0.4%).^25^ These results were similar to our findings. Therefore, internal iliac LLNs, as the gate point of lateral node metastasis, require more attention from clinicians.

Some previous studies had explored indicators that predicted LLNM or LLR based on MRI scan. The Lateral Node Study Consortium group had reported that >7 mm LLNs could lead to a significantly higher risk of LLR (19.5% 5‐year LLR rate).^3^ Kusters M et al. reported that the LLR rate was significantly higher in patients with LLNs >10 mm (33.3% 4‐year LLR rate).^26^ Kroon HM et al. also described patients with LLN malignant features (internal heterogeneity/border irregularity) had worse OS and DMFS.^10^ However, Kusters M et al. found that these malignant features were not associated with OS, LLR, and DMFS.^26^ Therefore, no unified conclusion can be reached by these studies. The second main finding from our study was that we developed a comprehensive LLNM risk scoring system, and we found that LLN risk scoring ≥2 could be considered as best predicted value for LLNM. It means that LLN with a short‐axis diameter >5 mm or with two malignant features or with a short‐axis diameter of 3–5 mm and one malignant feature could be considered malignant LLN. Patients with LLN risk scoring ≥2 had worse prognosis than those patients with LLN risk scoring <2, while better than those patients with SDM. This means that if those patients received timely treatment, they might have acceptable prognosis.

The third main finding was that the prognosis of patients with LLN > 10 mm tended to be similar to that in patients with SDM, while other patients with LLN <10 mm had relatively good prognoses. In one previous respective study, LLNM disease is not considered as a systemic disease but a regional disease, which could benefit from LLND.^18^, ^27^ Given the small number of patients in both group (LLN > 10 mm and SDM) and uneven baseline of treatment in this study, it is hard to conclude that LLN >10 mm could be considered to have distant metastasis disease. Well‐designed studies with larger sample size are required to further prove this finding.

Additionally, the Lateral Node Study Consortium group demonstrated that the median short‐axis size of the largest LLN on the primary MRI scan was 7.0 mm.^7^ In our cohort study, the largest LLN short‐axis diameter ranged from 3.85 mm to 5.41 mm. We found that 36.7 percent of patients had LLN short‐axis diameter <5 mm. Most patients developed measurable LLNs in the obturator cranial compartment, and the average number of LLNs was also the most (1.78) in this compartment. Canessa CE et al. reported the maximum number of LLNs was found in the obturator fossa, with a mean of 7 LLNs.^28^ However, in their study, the obturator fossa was not further subdivided into obturator cranial and caudal compartments. In our study, the mean LLNs in the whole obturator compartment was 6.27, roughly equivalent to the previous study. These results revealed that MDCT might be more capable of detecting small LLNs. Meanwhile, surgeons in our team pay more attention to lateral compartments, which also contributed to detecting so many LLNs.

In spite of our efforts to conduct the current study, it still has the following limitations. Firstly, this is a retrospective study; thus, selection bias could not be completely excluded. Secondly, differences in the doctors' experience and equipment might lead to the imbalances of baseline characteristics among groups. Thirdly, no histopathology was performed; therefore, it is unclear whether these enlarged LLNs were actually metastatic.

## 5 CONCLUSION

Our study comprehensively descripted the distribution characteristics and prognostic significance of LLNs. Our results confirmed that LLR is the main locoregional recurrence pattern. In addition, most rectal cancer patients have measurable LLNs. However, these patients with enlarged LLNs still have a significant better prognosis than patients with distant metastasis, which indicated the potential value of locoregional treatment for enlarged LLNs. Well‐designed studies with larger sample size are required to prove our results.

## AUTHOR CONTRIBUTIONS

**Xuyang Yang:** Conceptualization (equal); data curation (equal); formal analysis (equal); writing – original draft (equal). **Yang Zhang:** Conceptualization (equal); data curation (equal); formal analysis (equal); writing – original draft (equal). **Zixuan Zhuang:** Investigation (equal); methodology (equal). **Hanjiang Zeng:** Investigation (equal); methodology (equal); software (equal). **Tong Zhang:** Methodology (equal); project administration (equal); software (equal). **Xiangbing Deng:** Supervision (equal). **Wenjian Meng:** Methodology (equal); resources (equal); validation (equal). **Ziqiang Wang:** Funding acquisition (lead).

## FUNDING INFORMATION

This study was supported by the Department of Science and Technology of Sichuan Province (2023YFS0277; 2021YFS0025), 1·3·5 project for disciplines of excellence, West China Hospital, Sichuan University (20HXJS003); 1·3·5 project for disciplines of excellence‐Clinical Research Incubation Project, and West China Hospital, Sichuan University (22HXFH001; 2019HXFH031); Post‐Doctor Research Project, West China Hospital, Sichuan University (2021HXBH033); Post‐Doctor Research Project, Sichuan University (20826041E4084); China Postdoctoral Science Foundation (2022 M712264), the Ethicon Excellent in Surgery Grant (EESG) (No HZB‐20190528‐4).

## CONFLICT OF INTEREST STATEMENT

The authors declare that they have no conflicts of interest.

## ETHICS STATEMENT

Written informed consent was obtained from all participants, and this study was approved by the institutional ethical committee.

## Supporting information


Data S1.

## Data Availability

The original contributions presented in the study are included in the article/supplementary material; further inquiries can be directed to the corresponding author wangziqiang@scu.edu.cn.
