# Supplementary material for: Cross‐sectional investigation of the distribution characteristics and prognostic significance of lateral lymph nodes in patients with rectal cancer
Source: Cancer Med. 2024 Sep 23;13(18):e70170. doi: 10.1002/cam4.70170 (PMC11418819; doi:10.1002/cam4.70170)
Supplement: Supplementary file 1 — Data S1. [file CAM4-13-e70170-s001.docx]

**Table S1.** **The distribution regions of all observed lateral lymph nodes.**

| **Regions** | **No. of Cases Having Nodal Involvement, n** | **Total No. of Lymph Nodes Harvested, n** | **Average No. of Lymph Nodes Harvested, n** | **Short axis of the largest lymph node in short axis, mean + SD** | **Long axis of the largest lymph node in short axis, mean ± SD** |
| --- | --- | --- | --- | --- | --- |
| **L-CIC** | 163 | 268 | 1.64 | 4.63 ± 1.47 | 6.59 ± 5.11 |
| **R-CIC** | 74 | 95 | 1.28 | 4.20 ± 1.13 | 5.92 ± 1.70 |
| **L-EIC** | 76 | 99 | 1.30 | 4.69 ± 1.43 | 8.23 ± 3.73 |
| **R-EIC** | 101 | 142 | 1.41 | 4.63 ± 1.26 | 7.83 ± 3.72 |
| **L-OCRC** | 231 | 414 | 1.79 | 4.24 ± 1.41 | 6.79 ± 3.03 |
| **R-OCRC** | 281 | 539 | 1.92 | 4.53 ± 1.83 | 7.44 ± 5.76 |
| **L-OCAC** | 104 | 125 | 1.20 | 3.85 ±2.18 | 5.47 ± 2.99 |
| **R-OCAC** | 88 | 118 | 1.34 | 4.49 ± 2.35 | 7.29 ± 8.08 |
| **L- PIIC** | 18 | 20 | 1.11 | 5.42 ± 2.94 | 6.71 ± 2.91 |
| **R-PIIC** | 38 | 47 | 1.24 | 4.72 ± 3.43 | 6.37 ± 4.03 |
| **L- DIIC** | 49 | 61 | 1.24 | 5.11 ± 2.72 | 8.47 ± 11.34 |
| **R-DIIC** | 95 | 107 | 1.13 | 4.20 ± 1.76 | 5.62 ± 2.54 |
| **L-EDIIC** | 38 | 39 | 1.03 | 4.57 ± 2.03 | 6.38 ± 2.55 |
| **R- EDIIC** | 47 | 52 | 1.11 | 4.40 ± 2.09 | 5.91 ± 2.92 |

L-CIC, left common iliac compartment; R-CIC, right common iliac compartment; L-EIC, left external iliac compartment; R-EIC, right external iliac compartment; L-OCRC, left obturator cranial compartment; R-OCRC, right obturator cranial compartment; L-OCAC, left obturator caudal compartment; R-OCAC, right obturator caudal compartment; L-PIIC, left proximal internal iliac compartment; R-PIIC, right proximal internal iliac compartment; L-DIIC, left distal internal iliac compartment; R-DIIC, right distal internal iliac compartment; L-EDIIC, left extended distal internal iliac compartment; R-EDIIC, right extended distal internal iliac lymph node.

**Table. S2 The number and percentage of all measurable lateral lymph nodes according to the short axis and distribution regions.**

| **Regions** | **0-3mm** | **3-5mm** | **5-7mm** | **7-10mm** | **>10mm** |
| --- | --- | --- | --- | --- | --- |
| **L-CIC** | 11 (6.7) | 98 (60.1) | 45 (27.6) | 8 (4.9) | 1 (0.6) |
| **R-CIC** | 11 (14.9) | 48 (64.9) | 13 (17.6) | 2 (2.7) | 0 (0.0) |
| **L-EIC** | 7 (9.2) | 45 (59.2) | 19 (25.0) | 4 (5.3) | 1 (1.3) |
| **R-EIC** | 10 (9.9) | 50 (49.5) | 38 (37.6) | 3 (3.0) | 0 (0.0) |
| **L-OCRC** | 32 (13.9) | 149 (64.5) | 37 (16.0) | 12 (5.2) | 1 (0.4) |
| **R-OCRC** | 35 (12.5) | 155 (55.2) | 70 (24.9) | 15 (5.3) | 6 (2.1) |
| **L-OCAC** | 38 (36.5) | 47 (45.2) | 16 (15.4) | 2 (1.9) | 1 (1.0) |
| **R-OCAC** | 22 (25.0) | 39 (44.3) | 20 (22.7) | 5 (5.7) | 2 (2.3) |
| **L- PIIC** | 1 (5.6) | 8 (44.4) | 7 (38.9) | 1 (5.6) | 1 (5.6) |
| **R-PIIC** | 8 (21.1) | 22 (57.9) | 4 (10.5) | 2 (5.3) | 2 (5.3) |
| **L- DIIC** | 5 (10.2) | 28 (57.1) | 8 (16.3) | 5 (10.2) | 3 (6.1) |
| **R-DIIC** | 18 (18.9) | 57 (60.0) | 15 (15.8) | 3 (3.2) | 2 (2.1) |
| **L-EDIIC** | 5 (13.2) | 22 (57.9) | 7 (18.4) | 2 (5.3) | 2 (5.3) |
|  |  |  |  |  |  |
| **R- EDIIC** | 10 (21.3) | 26 (55.3) | 7 (14.9) | 3 (6.4) | 1 (2.1) |

L-CIC, left common iliac compartment; R-CIC, right common iliac compartment; L-EIC, left external iliac compartment; R-EIC, right external iliac compartment; L-OCRC, left obturator cranial compartment; R-OCRC, right obturator cranial compartment; L-OCAC, left obturator caudal compartment; R-OCAC, right obturator caudal compartment; L-PIIC, left proximal internal iliac compartment; R-PIIC, right proximal internal iliac compartment; L-DIIC, left distal internal iliac compartment; R-DIIC, right distal internal iliac compartment; L-EDIIC, left extended distal internal iliac compartment; R-EDIIC, right extended distal internal iliac lymph node.

**Table. S3 The number and percentage of patients with measurable lateral lymph nodes according to the short axis.**

| **0-3mm** | **3-5mm** | **5-7mm** | **7-10mm** | **>10mm** |
| --- | --- | --- | --- | --- |
| 23 (5.0) | 206 (44.9) | 158 (34.4) | 52 (11.3) | 20 (4.4) |
|  |  |  |  |  |

**Table.S4 The imaging characteristics of LLNs of patients with measurable LLNs.**

| **Characteristics** | **No.** |
| --- | --- |
| **The distribution** |  |
| Unilateral | 150 (32.7) |
| Bilateral | 309 (67.3) |
| **The value of CT** |  |
| Low（0-40） | 31 (18.8) |
| Middle（40-70） | 110 (24.0) |
| High（>70） | 318 (69.3) |
| **Morphology** |  |
| Regular | 296 (64.5) |
| Irregular | 163 (35.5) |
| **Texture** |  |
| Homogeneous | 347 (75.6) |
| Heterogeneous | 112 (24.4) |
| **Margin** |  |
| Clear | 266 (58.0) |
| Vague | 92 (20.0) |
| Nodular | 39 (8.5) |
| Burr-like | 62 (13.5) |

| **Short-axis** | **No measurable** | **0-3mm** | **3-5mm** | **5-7mm** | **7-10mm** | **>10mm** | **SDM** |
| --- | --- | --- | --- | --- | --- | --- | --- |
| **5-year OS** | 83.9 (76.3, 92.3) | 85.0 (70.7, 100.0) | 84.0 (78.8, 89.5) | 83.4 (77.3, 90.0) | 82.8 (72.0, 95.3) | 18.5 (5.3, 64.5) | 8.5 (3.3, 21.7) |
| **5-year DFS** | 83.7 (75.9, 92.2) | 80.0 (64.3, 99.6) | 82.7 (77.3, 88.4) | 80.0 (73.4, 87.2) | 81.2 (69.6, 94.8) | 20.0 (5.9, 67.3) | 0 |
| **5-year LRFS** | 98.8(96.4, 100.0) | - | 97.1 (94.7, 99.6) | 95.9 (92.5, 99.5) | 97.1 (91.5, 100.0) | 35.7 (13.4, 95.5) | 86.5 (75.3, 99.4) |
| **5-year LLRFS** | - | - | 97.6 (95.1, 1) | 95.2 (91.4, 990) | 97.1 (91.5,100.0) | 35.7(13.4, 95.5) | 93.6 (86.9, 100.0) |
| **5-year DMFS** | 84.9 (77.3, 93.2) | 80.0 (64.3, 99.6) | 83.1 (77.8, 88.8) | 82.3 (76.0, 89.2) | 84.1 (73.2, 96.7) | 22.5 (6.8, 74.1) | 0 |

**Table S5. The 5-year survival of patients with different LLNs in short axis.**

SDM, simultaneous distant metastasis; - represents no outcome event occurred; OS, overall survival; DFS, disease-free survival; LRFS, local recurrence-free survival; LLRFS, local lateral recurrence-free survival; DMFS, distant metastasis-free survival

**Table.S6. Univariate analyses of malignant score associated with the survival outcome.**

| **Variables** | **Overall survival** |  | **Local recurrence-free survival** |  | **Lateral recurrence-free survival** |  | **Distant metastasis-free survival** |  |
| --- | --- | --- | --- | --- | --- | --- | --- | --- |
|  | **HR (95% CI)** | ***P* value** | **HR (95% CI)** | ***P* value** | **HR (95% CI)** | ***P* value** | **HR (95% CI)** | ***P* value** |
| **Malignant score ≥ 1** | 1.643 (0.915, 2.948) | 0.096 | 4.631 (0.618, 34.693) | 0.136 | 28.760 (0.185, 4470.678) | 0.192 | 1.465 (0.846, 2.537) | 0.173 |
| **Malignant score ≥ 2** | 1.643 (1.081, 2.497) | 0.020 | 4.855 (1.415, 16.664) | 0.012 | 6.900 (1.578, 30.175) | 0.010 | 1.548 (1.031, 2.325) | 0.035 |
| **Malignant score ≥ 3** | 1.683 (1.122, 2.526) | 0.012 | 2.638 (1.072, 6.492) | 0.035 | 4.385 (1.621, 11.859) | 0.004 | 1.247 (0.826, 1.881) | 0.294 |
| **Malignant score ≥ 4** | 1.633 (1.022, 2.610) | 0.040 | 3.825 (1.538, 9.511) | 0.004 | 4.709 (1.817, 12.207) | 0.001 | 1.887 (0.720, 1.957) | 0.503 |
| **Malignant score ≥ 5** | 3.289 (1.922, 5.628) | 0.000 | 11.768 (4.724, 29.314) | 0.000 | 14.660 (5.642, 38.088) | 0.000 | 2.187 (1.196, 3.998) | 0.011 |
| **Malignant score ≥ 6** | 8.652 (4.461, 16.783) | 0.000 | 31.078 (11.467, 84.230) | 0.000 | 38.784 (13.890, 108.292) | 0.000 | 6.514 (3.142, 13.506) | 0.000 |
| **Malignant score ≥ 7** | 7.982 (2.519, 25.296) | 0.000 | 11.363 (1.502, 85.942) | 0.019 | 13.554 (1.777, 103.412) | 0.012 | 6.011 (1.475, 24.485) | 0.012 |

HR, hazard ratio
